# Clinical Profile of Children with Norovirus Disease in Rotavirus Vaccine Era

**DOI:** 10.3201/eid1910.130448

**Published:** 2013-10

**Authors:** Mary E. Wikswo, Rishi Desai, Kathryn M. Edwards, Mary Allen Staat, Peter G. Szilagyi, Geoffrey A. Weinberg, Aaron T. Curns, Benjamin Lopman, Jan Vinjé, Umesh D. Parashar, Daniel C. Payne, Aron J. Hall

**Affiliations:** Centers for Disease Control and Prevention, Atlanta, Georgia, USA (M.E. Wikswo, R. Desai, A.T. Curns, B. Lopman, J. Vinjé, U.D. Parashar, D.C. Payne, A.J. Hall);; Vanderbilt University Medical Center, Nashville, Tennessee, USA (K.M. Edwards);; Cincinnati Children’s Hospital Medical Center, Cincinnati, Ohio, USA (M.A. Staat);; University of Rochester School of Medicine and Dentistry, Rochester, New York, USA (P.G. Szilagyi, G.A. Weinberg)

**Keywords:** norovirus, rotavirus, viruses, norovirus disease, acute gastroenteritis, rotavirus vaccine, severity, children

**To the Editor:** After the substantial decrease in acute gastroenteritis (AGE) in children caused by rotavirus after introduction of 2 rotavirus vaccines (*1*), norovirus has become the leading cause of medically attended AGE in US children <5 years of age (*2*). We describe the clinical characteristics of norovirus disease and assessed whether rotavirus vaccine protected against norovirus AGE.

During October 2008–September 2010, the New Vaccine Surveillance Network enrolled 1,897 children <5 years of age with symptoms of AGE (≥3 episodes of diarrhea or any episodes of vomiting within 24 hours lasting ≤10 days) who came to hospitals, emergency departments, and outpatient clinics in Cincinnati, Ohio; Nashville, Tennessee; and Rochester, New York, USA, as described (*2*).

Epidemiologic, clinical, and vaccination data were systematically collected. Whole fecal specimens were obtained within 14 days of the date of visit and tested for rotavirus by using a commercial enzyme immunoassay (Rotaclone; Meridian Bioscience, Inc., Cincinnati, OH, USA) and for norovirus by using real-time reverse transcription quantitative PCR, followed by sequence analysis of positive samples (*3*,*4*). Clinical severity was assessed by using a 20-point scoring system (*5*), which was modified to use behavior as a proxy for dehydration. Odds ratios used to calculate vaccine effectiveness (VE) were adjusted for race and insurance status (online Technical Appendix, wwwnc.cdc.gov/EID/article/19/10/13-0448-Techapp1.pdf).

Inclusion criteria for this study corresponded with criteria used in previous New Vaccine Surveillance Network studies (*2*,*6*). Children were age eligible for pentavalent rotavirus vaccination (RV5), had a fecal specimen tested for norovirus and rotavirus, and had complete vaccination and AGE symptom information (Technical Appendix Figure 1). Children who received a dose of monovalent rotavirus vaccine or vaccine of unknown type or were positive for rotavirus and norovirus were excluded from analyses. Only unvaccinated rotavirus-positive children (n = 69, 72%) were used in severity score analyses because RV5 is known to attenuate rotavirus illness (*6*).

Of the enrolled children, 574 met the inclusion criteria; 144 (25%) norovirus-positive case-patients, 96 (17%) rotavirus-positive case-patients, and 334 (58%) patients negative for norovirus and rotavirus (control patients with AGE) (Technical Appendix Figure 1). Of 144 norovirus-positive specimens, 10 (7%) could not be genotyped, 4 (3%) were positive for genogroup (G) I, and 130 (90%) were positive for GII. The most common genotype was GII.4 Minerva (74 [51%]).

Norovirus case-patients were significantly more likely than control patients with AGE to have longer duration and more episodes of vomiting in a 24-hour period (p = 0.003 and p<0.0001, respectively) but were significantly less likely to report fever (p = 0.001) (Table). However, the median severity score for norovirus case-patients did not differ from that for control patients with AGE (11 vs. 10, respectively). Individual severity score components and overall severity scores did not differ among case-patients infected with norovirus who received 0, 1 or 2, or 3 doses of RV5, but the duration of vomiting was longer in case-patients infected with norovirus GII.4 than in those infected with a non-GII.4 genotype (Technical Appendix Tables 1, 2; Figure 2).

**Table:** Clinical profile and severity score of norovirus case-patients compared with acute gastroenteritis control patients and unvaccinated rotavirus case-patients, New Vaccine Surveillance Network, United States, 2008–2010*

Severity score component	Severity score	Norovirus case-patients, n = 144	Unvaccinated rotavirus case-patients, n = 69	p value†	AGE control patients, n = 334	p value†
Duration of diarrhea, d, no. (%)				**0.003**		0.19
0	0	32 (22)	3 (4)		82 (25)	
1–4	1	87 (60)	55 (80)		171 (51)	
5	2	13 (9)	7 (10)		33 (10)	
≥6	3	12 (8)	4 (2)		48 (14)	
Diarrhea episodes/24 h, no. (%)				**0.0003**		0.24
0	0	32 (22)	3 (4)		82 (25)	
1–3	1	47 (33)	16 (23)		79 (24)	
4–5	2	22 (15)	14 (20)		64 (19)	
≥6	3	43 (30)	36 (52)		23 (33)	
Duration of vomiting, h, no. (%)				0.43		**0.003**
0	0	7 (5)	2 (3)		54 (16)	
1–23 (1 d)	1	28 (19)	10 (14)		64 (19)	
24–47 (2 d)	2	33 (23)	12 (17)		74 (22)	
≥48 (≥3 d)	3	76 (53)	45 (65)		142 (43)	
Vomiting episodes/24 h, no. (%)				0.22		**<0.0001**
0	0	7 (5)	2 (3)		54 (16)	
1	1	11 (8)	1 (1)		52 (16)	
2–4	2	45 (31)	20 (29)		117 (35)	
≥5	3	81 (56)	46 (67)		111 (33)	
Fever, °F, no. (%)				**<0.0001**		**<0.0001**
≤98.6	0	80 (56)	15 (22)		102 (31)	
98.7–101.1	1	29 (20)	21 (30)		55 (16)	
101.2–102	2	9 (6)	18 (26)		45 (13)	
≥102.1	3	26 (18)	15 (22)		132 (40)	
Signs, no. (%)				**<0.0001**		0.65
Normal	0	12 (8)	2 (3)		35 (10)	
Less playful/irritable	1	63 (44)	13 (19)		158 (47)	
Lethargic/listless	2	67 (47)	54 (78)		138 (41)	
Seizure	3	2 (1)	0 (0)		3 (1)	
Treatment, no. (%)				**0.02**		0.16
None	0	50 (35)	12 (17)		135 (40)	
Rehydration, no hospitalization	1	50 (35)	26 (38)		87 (26)	
Hospitalization	2	44 (31)	31 (45)		112 (34)	
Severity score, median	NA	11	13	**<0.0001**	10	0.78

*AGE control patients with acute gastroenteritis (defined ≥3 more episodes of diarrhea or any episodes of vomiting within 24 h that lasted ≤10 d), but who were negative for norovirus and rotavirus. NA, not applicable. †Severity scores were compared by Wilcoxon rank-sum test. All other components were compared by Fisher χ^2^ test. Significant findings are indicated in **boldface**.

Relative to the 69 unvaccinated rotavirus case-patients, norovirus case-patients had shorter duration and fewer episodes of diarrhea in a 24-hour period (p = 0.003 and p = 0.0003, respectively). Norovirus case-patients were also significantly less likely to be hospitalized (p = 0.02), have fever (p<0.0001), and have severe behavior changes (p<0.0001); they also had lower overall severity scores (p<0.0001) than unvaccinated rotavirus case-patients.

Compared with control patients with AGE, VE of any dose of RV5 against norovirus disease was −0.9% (95% CI −55% to 34%). A full course of RV5 likewise showed no evidence of protection against norovirus (VE 5%; 95% CI −50% to 40%), and results were consistent across age groups.

In conclusion, we found that norovirus AGE was associated with more frequent and prolonged vomiting but less fever than AGE not caused by norovirus or rotavirus. Case-patients infected with norovirus GII.4 also had a longer duration of vomiting than did case-patients infected with non-GII.4 norovirus genotypes. However, AGE among unvaccinated rotavirus case-patients was more severe than among norovirus case-patients, and was characterized by higher fever and more frequent and severe diarrhea. This finding confirms findings in a study of children in Finland (*7*), although our study found no difference in frequency or severity of vomiting between patients with rotavirus disease or those with norovirus disease.

In addition, vaccination against rotavirus did not provide protection against norovirus and had no effect on the clinical course of norovirus disease, which is consistent with other findings (*8*). Although an earlier rotavirus vaccine, which has subsequently been withdrawn, may have provided some nonspecific protection by reducing intensity and duration of diarrhea associated with adenovirus and sapovirus (*9*,*10*), our study did not demonstrate a similar effect on norovirus-associated diarrhea after vaccination with RV5. This study reinforces the hypothesis that norovirus can cause severe AGE among young children and should be considered as a specific target for vaccine development.

Technical Appendix Detailed information on clinical profile of children with norovirus disease in rotavirus vaccine era.
