# Supplementary material for: Clinical Profile of Children with Norovirus Disease in Rotavirus Vaccine Era
Source: Emerg Infect Dis. 2013 Oct;19(10):1691–3. doi: 10.3201/eid1910.130448 (PMC3810752; doi:10.3201/eid1910.130448)
Supplement: Technical Appendix — Detailed information on clinical profile of children with norovirus disease in rotavirus vaccine era. [file 13-0448-Techapp-s1.pdf]

Article DOI: http://dx.doi.org/10.3201/eid1910.130448

Clinical Profile of Children with Norovirus Disease in Rotavirus Vaccine Era

Technical Appendix

Detailed Information on Clinical Profile of Children with Norovirus Disease in Rotavirus Vaccine Era

Methods

Persons included in the study were enrolled as New Vaccine Surveillance Network study participants (n = 1,897) and had a fecal specimen collected (n = 1,363, 72%) that was tested for norovirus and rotavirus (n = 1,295, 95%) (Technical Appendix Figure 1). Pentavalent rotavirus vaccine (RV5) (RotaTeq; Merck and Co. Inc., Whitehouse Station, NJ, USA) was recommended for routine use in US infants in February 2006 in children 6–32 weeks of age. Therefore, to best gauge the possible effect of rotavirus vaccination on acute gastroenteritis (AGE) in this study population, we further restricted study participants to those born on or after April 1, 2006 (n = 1,178), and who had reached the Advisory Committee on Immunization Practices–recommended age for completion of RV5 vaccine series (i.e., ≥8 months of age), to avoid confounding by age at time of last dose (n = 759) (*1*). Furthermore, only those children for whom a complete provider-verified vaccination record could be obtained were included (n = 748). Only rotavirus vaccination doses administered >14 days before AGE symptom onset were included. Study participants receiving any dose of monovalent rotavirus vaccine (Rotarix; GlaxoSmithKline Biologicals, Rixensart, Belgium) or a dose of unknown type were further excluded (n = 677). To ensure a complete severity analysis, only persons with complete severity score data were included in final analysis (n = 577). Of the remaining persons, 144 (25%) were positive for norovirus, 96 (17%) were positive for rotavirus, and 334 (58%) were negative for norovirus and rotavirus. Three persons positive for norovirus and rotavirus and were excluded from analysis.

Demographic characteristics of norovirus-positive case-patients and control patients with AGE were assessed for potential confounding of comparisons in clinical profile. Vaccine effectiveness (VE) of a full course and any dose of rotavirus vaccine were calculated at (1 – adjusted odds ratio) × 100; odds ratios were adjusted for race and insurance status on the basis of results of the analysis of demographic characteristics.

Results

Current or past breastfeeding, premature birth, household size, highest household degree, and daycare attendance did not differ between norovirus case-patients and control patients with AGE (Technical Appendix Table 1). However, control patients with AGE were significantly more likely to be black, non-Hispanic ($p = 0.02$), and use public insurance ($p = 0.01$) than were norovirus case-patients. These groups may have been less likely to seek care for norovirus AGE given the lower occurrence of fever compared with other causes of AGE. Despite these differences, control patients with AGE have been found to be the most appropriate control group during other New Vaccine Surveillance Network studies (*2*,*3*).

Of 144 norovirus-positive specimens, 134 (93%) could be genotyped. Of these, 89 (66%) were positive for genogroup II type 4 (GII.4). Forty-one (31%) were positive for a GII genotype other than GII.4, and 4 (3%) were positive for GI. Of the 45 non-GII.4 genotypes found, GII.12 was the most common (n = 19, 46%). Case-patients with norovirus GII.4 reported a significantly longer duration of vomiting than patients with non-GII.4 norovirus ($p = 0.05$), but the median overall severity score (11) for each group was the same (Technical Appendix Table 2).

To validate our results for vaccine effectiveness (VE) of RV5 against norovirus disease, we also calculated VE against rotavirus disease. VE of RV5 against rotavirus disease was 84% (95% CI 73%–90%) for any dose and 86% (95% CI 74% – 92%) for a full course, consistent with other recent studies (*2*–*4*). Furthermore, we compared individual severity score components and overall severity scores among norovirus case-patients receiving 0, 1 or 2, or 3 doses of RV5 (Technical Appendix Figure 2). There were no differences in any severity score components when case-patients with norovirus receiving 3 doses of RV5 were compared with to unvaccinated case-patients with norovirus, or when norovirus case-patients receiving 1 or 2

doses were compared with unvaccinated case-patients with norovirus. Median severity scores among each of these groups of case-patients with norovirus were likewise not different.

References

1. Cortese MM, Partashar UD; Centers for Disease Control and Prevention. Prevention of rotavirus gastroenteritis among infants and children: recommendations of the Advisory Committee on Immunization Practices (ACIP). MMWR Recomm Rep. 2009;58:1–25. PubMed

2. Payne DC, Boom JA, Staat MA, Edwards KM, Szilagyi PG, Klein EJ, et al. Effectiveness of pentavalent and monovalent rotavirus vaccines in concurrent use among US children <5 years of age, 2009–2011. Clin Infect Dis. 2013;57:13–20. PubMed http://dx.doi.org/10.1093/cid/cit164

3. Donauer S, Payne DC, Edwards KM, Szilagyi PG, Hornung RW, Weinberg GA, et al. Determining the effectiveness of the pentavalent rotavirus vaccine against rotavirus hospitalizations and emergency department visits using two study designs. Vaccine. 2013; 31:2692–7. PubMed http://dx.doi.org/10.1016/j.vaccine.2013.03.072

4. Staat MA, Payne DC, Donauer S, Weinberg GA, Edwards KM, Szilagyi PG, et al. Effectiveness of pentavalent rotavirus vaccine against severe disease. Pediatrics. 2011;128:e267–75. PubMed http://dx.doi.org/10.1542/peds.2010-3722

Technical Appendix Table 1. Characteristics of norovirus case-patients and acute gastroenteritis control patients, New Vaccine Surveillance Network United States, 2008–2010*

Characteristic	Norovirus case-patients, n = 144	Control patients with AGE, n = 334	p value†
Median age (range), mo	16.5 (8–46)	18 (8–49)	0.06
Sex, no. (%)			
M	74 (51)	188 (56)	0.37
F	70 (49)	146 (44)	
Race, no. (%)			
White, non-Hispanic	63 (44)	119 (36)	**0.02**
Black, non-Hispanic	44 (31)	146 (44)	
Hispanic	30 (21)	47 (14)	
Other/unknown	7 (5)	22 (7)	
Insurance, no. (%)			
Public or public/private	93 (65)	257 (77)	**0.01**
Private	45 (31)	63 (19)	
None/unknown	6 (4)	14 (4)	
Breastfeeding, no. (%)			
Never	53 (37)	124 (37)	0.32
Past	86 (60)	184 (55)	
Present	5 (3)	25 (7)	
Unknown	0 (0)	1 (<1)	
Premature birth, no. (%)			
No	127 (88)	297 (89)	0.88
Yes	17 (12)	35 (10)	
Unknown	0 (0)	2 (1)	
Daycare attendance, no. (%) children			
None	101 (70)	199 (60)	0.07
<6	16 (11)	37 (11)	
6–12	20 (14)	75 (22)	
>12	7 (5)	16 (5)	

Characteristic	Norovirus case-patients, n = 144	Control patients with AGE, n = 334	p value†
Unknown	0 (0)	7 (2)	
Highest degree achieved by any household member, no. (%)			0.55
None	16 (11)	35 (10)	
GED	9 (6)	26 (8)	
High school diploma	65 (45)	171 (51)	
2–4 y of college	37 (26)	71 (21)	
Graduate	12 (8)	26 (8)	
Unknown	5 (3)	5 (2)	
Household size, no. persons (%)			
2–4	92 (64)	211 (63)	0.61
5–7	44 (31)	108 (32)	
≥8	8 (6)	12 (4)	
Unknown	0 (0)	3 (1)	
RV5‡ vaccination, no. (%)			
Not vaccinated	42 (29)	98 (29)	0.79
Partial (1 or 2 doses)	72 (50)	175 (52)	
Full course (3 doses)	30 (21)	61 (18)	

*AGE, acute gastroenteritis; GED, general educational development; RV5, pentavalent rotavirus vaccine. Control patients with AGE had acute gastroenteritis defined as ≥3 episodes of diarrhea or any episodes of vomiting within 24 h lasting ≤10 d, but were negative for norovirus and rotavirus.
†Ages were compared by Wilcoxon rank-sum test. All other characteristics were compared by Fisher χ^2 test. Significant findings are indicated in **boldface**.
‡RotaTeq (Merck and Co. Inc., Whitehouse Station, NJ, USA).

Technical Appendix Table 2. Clinical profile and severity score of case-patients infected with norovirus with GII.4 compared with case-patients infected with a norovirus non-GII.4 genotype, New Vaccine Surveillance Network, United States, 2008–2010*

Severity score component	Severity score	Norovirus GII.4 case-patients, n = 89	Norovirus non-GII.4 case-patients, n = 45	p value†
Duration of diarrhea, d, no. (%)				0.06
0	0	16 (18)	15 (33)	
1–4	1	53 (60)	26 (58)	
5	2	12 (13)	1 (2)	
≥6	3	8 (9)	3 (7)	
Diarrhea episodes/24 h, no. (%)				0.07
0	0	16 (18)	15 (33)	
1–3	1	30 (34)	12 (27)	
4–5	2	11 (12)	9 (20)	
≥6	3	32 (36)	9 (20)	
Duration of vomiting, h, no. (%)				**0.05**
0	0	3 (3)	2 (4)	
1–23 (1 d)	1	16 (18)	10 (22)	
24–47 h (2 d)	2	14 (16)	15 (33)	
≥48 h (≥3 d)	3	56 (63)	18 (40)	
Vomiting episodes/24 h, no. (%)				1.0
0	0	3 (3)	2 (4)	
1	1	7 (8)	3 (7)	
2–4	2	28 (31)	14 (31)	
≥5	3	51 (57)	26 (58)	
Fever, °F, no. (%)				0.06
≤98.6	0	49 (55)	28 (62)	
98.7–101.1	1	22 (25)	4 (9)	
101.2–102	2	3 (3)	5 (11)	
≥102.1	3	8 (18)	15 (17)	
Signs, no. (%)				0.45
Normal	0	6 (7)	6 (13)	
Less playful/irritable	1	37 (42)	20 (44)	
Lethargic/listless	2	44 (49)	19 (42)	
Seizure	3	2 (2)	0 (0)	
Treatment, no. (%)				0.97
None	0	28 (31)	15 (33)	
Rehydration, no hospitalization	1	34 (38)	16 (36)	
Hospitalization	2	27 (30)	14 (31)	
Severity score, median	NA	11	11	0.12

*Significant findings are indicated in **boldface**. NA, not applicable.
†Severity scores compared by Wilcoxon rank-sum test. All other components compared by Fisher χ^2 test.

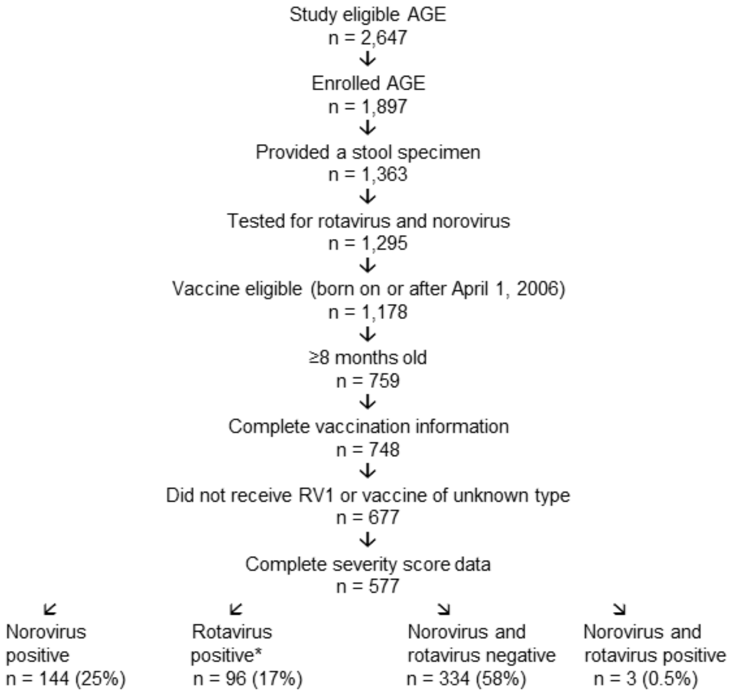

Technical Appendix Figure 1. Inclusion and exclusion criteria for patients with acute gastroenteritis (AGE), New Vaccine Surveillance Network, United States, 2008–2010. *69 (72%) of rotavirus-positive persons with AGE were not vaccinated against rotavirus; 27 (28%) received ≥1 dose pentvalent rotavirus vaccine (RV5). RV1 monovalent rotavirus vaccine.

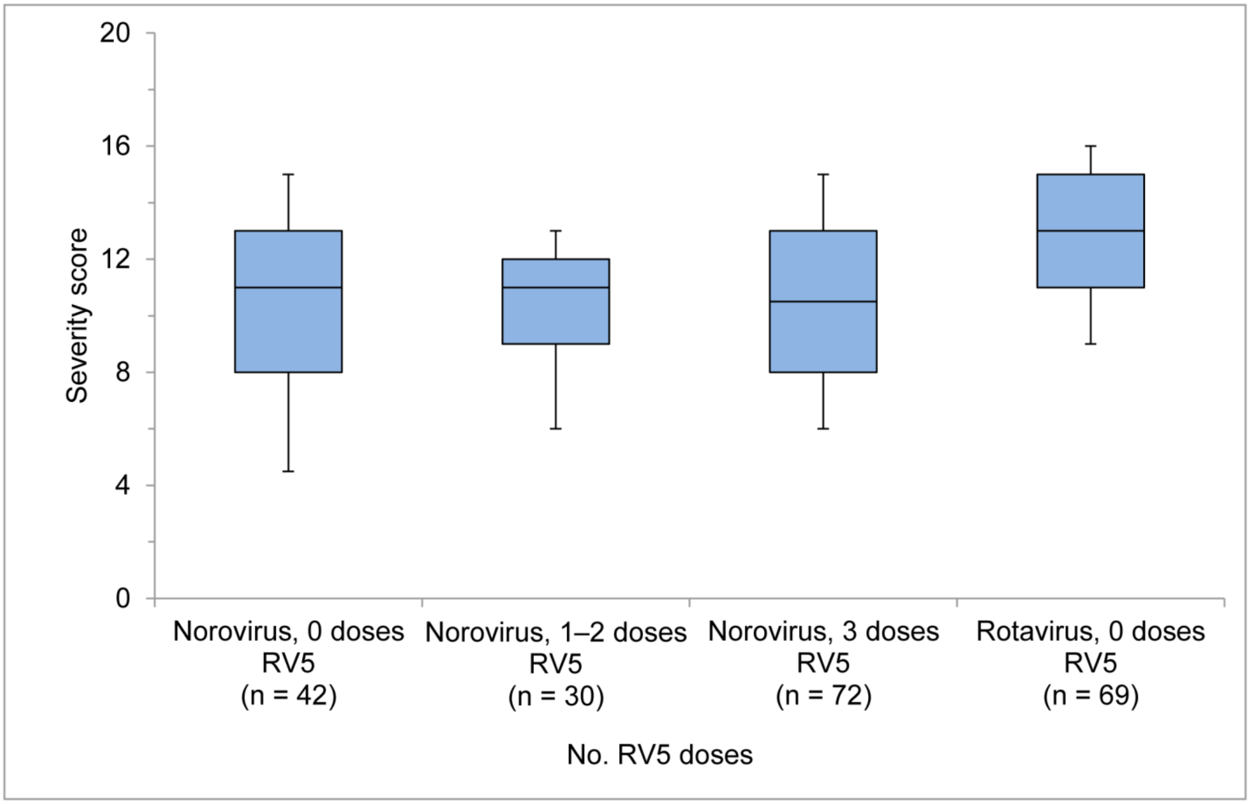

Technical Appendix Figure 2. Clinical severity scores among norovirus and rotavirus case-patients by no. pentavalent rotavirus vaccine (RV5) doses received, New Vaccine Surveillance Network, United States, 2008–2010. Horizontal lines indicate medians, error bars indicate interquartile ranges, and the minimum and maximum severity score values for each group. Rotavirus case-patient severity scores were significantly higher than those for each norovirus case-patient group by Wilcoxon rank-sum test ($p<0.05$, for all comparisons).
